# The data used to build the models: Pertussis morbidity and mortality burden considering various Brazilian data sources

**DOI:** 10.1016/j.vaccine.2020.09.007

**Published:** 2021-01-03

**Authors:** Angela M. Bagattini, Gabriela Policena, Ruth Minamisava, Ana Lucia S. Andrade, Sérgio de A. Nishioka, Anushua Sinha, Louise B. Russell, Cristiana M. Toscano

**Affiliations:** a Institute of Tropical Pathology and Public Health, Federal University of Goiás, Goiânia, Goiás, Brazil; b School of Nursing, Federal University of Goiás, Goiânia, Goiás, Brazil^1^; c Institute of Tropical Pathology and Public Health, Federal University of Goiás, Goiânia, Goiás, Brazil^2^; d National Coordination of Transmittable Diseases Surveillance, Secretary of Health Surveillance (SVS), Brazilian Ministry of Health, Brazil^3^; e Department of Medicine, Division of Infectious Diseases, Rutgers New Jersey Medical School, Newark, NJ, USA; f University of Pennsylvania, Department of Medical Ethics and Health Policy, 423 Guardian Drive, Philadelphia, PA 19104, USA^4^

**Keywords:** Pertussis, Whooping cough, Epidemiology, Surveillance, Mortality, Disease disease burden, Modelling

## Abstract

**Background:**

Pertussis is associated with significant disease burden in children worldwide. In addition to its cyclical nature, resurgences of pertussis cases, hospitalizations and deaths have been reported by many countries. We describe the dynamics of pertussis in Brazil, a middle-income country that has experienced a resurgence and that provides good quality data to allow building a dynamic transmission disease model.

**Methods:**

We conducted a descriptive analysis of pertussis burden considering data from the national disease surveillance system, national hospitalization information system and national mortality registry. Study period was 2000–2016. Absolute numbers and rates per 100,000 inhabitants over time, by age sub-groups and geographical regions are presented.

**Results:**

From 2000 to 2016, a total of 37,299 reported pertussis cases, 25,240 hospitalizations, and 601 deaths due to pertussis were reported. Although the outcomes – pertussis cases, hospitalizations, and deaths – come from independent information systems, our results document low disease burden with periodic increases every 3–4 years during the years 2000–2010, followed by a sharp increase which peaked in 2014. In both periods, disease burden is concentrated in young children, while its more serious outcomes – hospitalizations and deaths, are concentrated in infants. Pre-outbreak and outbreak disease burden as well as timing of peak during the outbreak period vary by states and within geographical regions, representing valuable resources of data for modelling purposes.

**Conclusion:**

Consistent disease burden patterns were observed over time in Brazil using a variety of data sources. Given the scarcity of good epidemiological data on pertussis available from low- and middle-income countries, our reported data provide valuable information for the assessment of the public health impact and cost-effectiveness modelling studies of newer strategies to prevent and control pertussis. These data were used to build and calibrate a national dynamic transmission model, which was used to evaluate the cost-effectiveness of maternal immunization.

Clinical Trial registry name and registration number: Not applicable.

## 1 Introduction

Pertussis disease burden is significant with recent estimates pointing at 24·1 million pertussis cases and 160 700 deaths from pertussis in children younger than 5 years of age globally in 2014. Developing countries, particularly in the African region, contribute the most to this burden. Pertussis mortality is particularly important in infants younger than 1 year of age [1].

Evidence points to a recent increase in cases of pertussis in addition to the naturally occurring cyclic disease pattern. Several are the hypotheses that have been raised to explain these outbreaks including improvements of laboratory diagnosis and sensitivity of surveillance systems, variable vaccine efficacy, and reduction of vaccination coverage or delays in the administration of the primary vaccination schedule [2].

The observed outbreaks and increases in pertussis cases and deaths have led public health authorities to consider new pertussis control strategies, such as maternal immunization and cocooning, among others. Analyses of such strategies requires robust data to support public health decision making as well as health impact and cost-effectiveness models. These data may be scarce in low- and middle-income countries (LMICs), where the needs may be greatest.

Brazil, a middle-income country, has collected good-quality data from a variety of sources for a long period of time. Like many other LMICs, Brazil has experienced a recent outbreak of pertussis and uses whole cell pertussis (wP) vaccine in its routine childhood immunization program. Together with the considerable within-country variation of socio-economic and epidemiologic conditions, these facts suggest that Brazilian pertussis morbidity and mortality data, at the national and sub-national levels, can be used to represent the dynamics of the disease in other LMICs as well.

Thus, the aim of this study is to describe the burden of pertussis in a middle-income country with good-quality morbidity and mortality information systems over a long period of time and in which an outbreak of pertussis has been experienced. The data presented were used to build and calibrate a national dynamic transmission model, which was used to evaluate the cost-effectiveness of maternal immunization in Brazil and in two low-income countries[3], and three state dynamic transmission models, which tested whether a single model structure could describe pertussis in different socioeconomic settings [4]. This study describes pertussis cases, hospitalizations and deaths reported to various information systems in Brazil, from January 2000 to December 2016.

## 2 Methods

### 2.1 Study design, period and setting

This is an observational descriptive study of pertussis trends over time, by age sub-groups and across geographical areas of the country with diverse socioeconomic conditions and vaccine coverage levels. Study period was 2000 to 2016, and included Brazil as a whole and each of its 27 federal units (herewith denominated states).

Brazil is an upper middle-income country with more than 200 million inhabitants, being thus the 6th most populous country in the world. Occupying a vast geographical area (fifth largest country in the world), it is divided in 5 macro-regions, 27 states and 5570 municipalities [5] (Supplementary Fig. 1). With a current national human development index (HDI) of 0.759 (2017), significant sub-national variation of socio-economic, demographic and development indicators are observed (Supplementary Table 1) [6]**.** As such, different realities can be explored, taking the advantage of available high-quality health data sources and national registries over a long period of time.

As in most developing countries in the world, wP vaccine was introduced in Brazil in the 70s. Following global recommendations to administer a 3-dose primary series initiated at 6 and no later than 8 weeks of age [2], wP is administered as a three-dose schedule at 2, 4 and 6 months of age plus 2 booster doses at 15 months and 48 months of age. Initially trivalent vaccine containing pertussis, diphtheria and tetanus antigens was administered [7]. In 2002 this vaccine was replaced by tetravalent vaccine (trivalent + *Haemophilus influenzae* type b antigen), which was in 2012 further replaced by a pentavalent vaccine (tetravalent + hepatitis B antigen). In late 2014 maternal vaccination with acellular pertussis (ap) vaccine targeting pregnant women from 27 weeks of gestation was introduced into the national routine immunization schedule by replacing the previous Td vaccine for pregnant women with Tdap.

### 2.2 Data sources

We considered pertussis morbidity and mortality data obtained from the following national information systems in Brazil: the National Notifiable Disease Information System (SINAN)[8], the Hospitalization Information System (SIH-SUS)[9], and the Mortality Information System (SIM)[10], as described below in further detail [11]. These are separate data systems and thus provide independent information about trends and patterns in pertussis burden. National and state level data were analyzed.

#### 2.2.1 Reported cases (SINAN)

Individual case by case information for all suspected pertussis cases reported by all municipalities in the country is available for the study period through SINAN, the Brazilian National Notifiable Disease Information System [8].

A case was defined as any individual, regardless of age or vaccination status, presenting with dry cough for at least 14 days, and either 1) one of the following symptoms: paroxysms of many, rapid coughs followed by a high-pitched “whoop” sound, inspiratory stridor, post-cough vomiting, cyanosis, apnea or asphyxia; or 2) contact with confirmed case [7]. Suspected cases can be laboratory confirmed when either a culture or real-time -polymerase chain reaction (qPCR) are positive. When samples are not collected or not adequate, cases can also be confirmed by epidemiologic criteria (suspected case who had contact with a lab-confirmed case during its infectious period), or clinical compatibility (presence of at least 2 pertussis-related symptoms). As such, criteria for case confirmation of cases reported to the surveillance system are indicated after adequate surveillance investigation. Laboratory confirmation by qPCR was implemented, starting in 2010, only in selected states in reference laboratories and at the national reference laboratory [11]. Laboratory is the most specific criterion for case confirmation, as very few false-positive cases will be confirmed (but more false-negative cases will occur). Clinical confirmation is the most sensitive criterion as very few false-negative cases will be confirmed (but more false-positive cases will occur). In 2014, the suspected case definition for children <6 months was revised, becoming more sensitive to cough duration; at least 10 days of any type of cough replaced the previous case definition for this age group (14 days of dry cough) [12].

Case by case surveillance data on all pertussis cases reported to the National Notifiable Disease Information System (SINAN) [8] during the study period, without personal identifiers, was made available by the National Coordination of Respiratory Transmitted Diseases Surveillance at the Secretary of Health Surveillance of the Brazilian Ministry of Health (SVS/MoH). We report on all cases confirmed by any criterion. It should be noted that cases reported to the surveillance system include all cases, regardless of outcome, so in addition to pertussis outpatients, hospitalized cases and cases who died due to pertussis are also expected to be reported to this information system.

#### 2.2.2 Hospitalizations (SIH-SUS)

The National Hospitalization Information System (SIH-SUS) collects individual level information on all hospitalizations occurring in the National Public Health System in Brazil, which provides services to approximately 75% of the country’s population, although the percentage covered varies by state and through time [13].

Cases were defined as any hospitalization occurring in the SIH-SUS in which the main discharge diagnosis was pertussis, as coded by the International Statistical Classification of Diseases and Related Health Problems, 10th revision (ICD-10) which has been used in Brazil since 1996 (ICD-10 code: A37). The main diagnosis is defined as a primary cause of hospitalization [14].

All pertussis hospitalizations recorded in the SIH-SUS during the study period were obtained online through the Ministry of Health DATASUS data web portal [9], by month, year, and state. Individual data files for 2000–2016, zipped in .DBC format, were downloaded in April 2018, and unzipped using TABWIN/DATASUS. Date of birth, date of hospitalization, place of residence, place of hospitalization, and outcomes for each case were obtained.

We also obtained vaccine coverage data from the National Vaccine Coverage Information System (SI-PNI) for both children (DTP3 coverage in children <1 year of age) and maternal Tdap vaccine coverage (which was introduced in Brazil in late 2014) and report data by state and year during the study period. [15]

Extracted databases were then merged into a single file using Stata v. 13.0 (Stata Corporation, Texas, USA).

#### 2.2.3 Deaths (SIM)

The National Mortality Information System (SIM) includes individual level information on all deaths registered in the country, including cause of death (by ICD code) and dates of birth and death, among others [10]. Pertussis-related deaths were defined as any death in which the main cause of death was pertussis, as coded by the ICD-10 (ICD10 code: A37). Data from all pertussis deaths recorded in SIM during the study period, without personal identifiers, were made available by the Department of Health Information Analysis (DASIS) of the Brazilian Ministry of Health.

#### 2.2.4 Population (IBGE)

To calculate rates, population data from the 2000 and 2010 National population censuses for the country and each of the states were obtained from the Brazilian Institute of Geography and Statistics (IBGE) [16]. For the inter-census period, population by age group for the country as a whole and each of its 27 states were estimated for all years through arithmetic interpolation between the two known points - the 2000 and 2010 censuses. The following age groups were considered: < 5 years, 5–9 years, 10–14 years, 15–19 years, 20–59 years, and 60 years and older. Children under 5 years were further disaggregated by the following age subgroups: <2, 2–3, 4–5, 6–23, and 24–59 months of age.

### 2.3 Statistical analysis

The absolute (numbers) and relative frequency (%) of confirmed cases, hospitalizations, and deaths, for each year for the country and by age groups are reported. Age of each reported case, hospitalization, or death was calculated from the reported date of birth and the date of disease onset, hospitalization, or death, respectively. Cases for which age could not be calculated were excluded from the analysis. Annual incidence rates, hospitalizations, and pertussis deaths per 100,000 inhabitants were calculated by age and year. Geographic distribution of cases and rates by macro-region of the country and states over time and across age groups is described.

For the distribution of event over time we considered two time-periods: pre-outbreak (2000–2010), and outbreak (2011–2016) periods. Mean incidence, hospitalization and mortality rates were estimated for the pre-outbreak and outbreak periods, and compared by period, age groups, and macro-region of the country.

We used t-tests to compare continuous variables and chi-square tests to compare proportions, considering a 0.05 significance level. Statistical analysis was performed with IBM SPSS version 23. Terra View 4.2.2 software updated on 2018/10/06 was used to map the distribution of pertussis incidence in space and time [17].

### 2.4 Ethical aspects

The project was approved by the Research Ethics Committee of the Clinical Hospital of the Federal University of Goiás (UFG) (Project # CAAE: 44817015.5.0000.5083; Approval # 1,098,718). Personal identifiers were not obtained from any data source.

## 3 Results

**Fig. 1:**
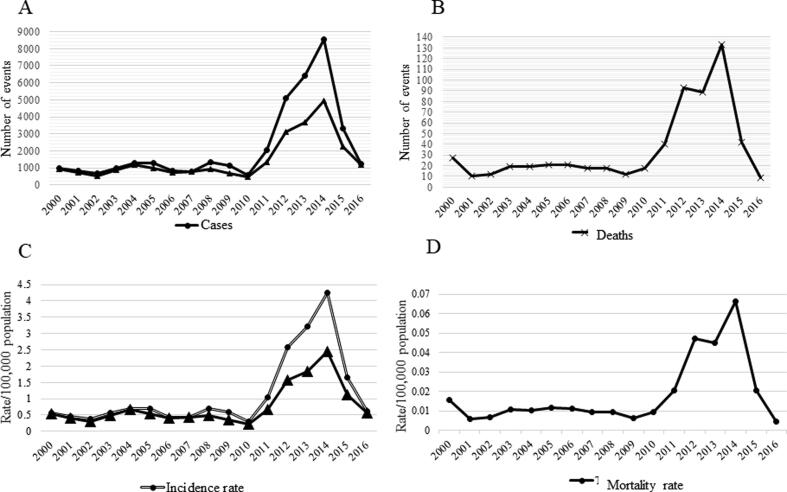
(A) Pertussis cases and hospitalizations over time. (B) Pertussis deaths over time. (C) Pertussis incidence and hospitalization rates per 100,000 population. (D) Pertussis mortality rate per 100,000 population. Brazil, 2000–2016.

Although the outcomes – pertussis cases, hospitalizations, and deaths – come from independent information systems, they all document two features: low disease burden, with periodic increases every 3–4 years during the years 2000–2010, followed by a sharp increase in events which peaked in 2014 (Fig. 1), and; concentration of disease, especially its more serious outcomes, in infants (Table 1).

**Table 1:** Frequency of pertussis reported cases, hospitalizations and deaths, by age group. Brazil, 2000–2016.

Age Groups	Cases N (%)	Hospitalization N (%)	Deaths N (%)
<2 months	7198 (19)	7860 (31)	290 (48)
2–3 months	8798 (24)	8595 (34)	237 (39)
4–5 months	4107 (11)	3464 (14)	38 (6)
6–11 months	3195 (9)	2188 (9)	21 (3)
12–23 months	2156 (6)	1084 (4)	7 (1)
2–4 years	3640 (10)	951 (4)	1 (0)
5–9 years	3145 (8)	498 (2)	0 (0)
10–14 years	1635 (4)	210 (1)	0 (0)
15–19 years	661 (2)	66 (0)	0 (0)
20–59 years	2650 (7)	201 (1)	2 (0)
60+ years	114 (0)	123 (0)	5 (1)

**Overall**	**37.299 (1 0 0)**	**25.240 (1 0 0)**	**601 (1 0 0)**

### 3.1 Pertussis burden over time

Between 1 January 2000 and 31 December 2016, a total of 38,319 confirmed pertussis cases were reported to SINAN, of which 1020 were excluded from this analysis as we could not estimate their age, thus resulting in 37,299 cases analyzed. A total of 25,240 pertussis hospitalization reported to SIH-SUS, and a total of 601 pertussis deaths registered in SIM were analyzed. (Fig. 1, panels A and B)

Annual incidence rates based on confirmed cases reported to SINAN, hospitalization rates based on pertussis cases hospitalized in the SUS, and mortality rates based on deaths due to pertussis are presented in Fig. 1 (panels C and D). Rates reflect the same pattern of distribution of case counts over time, but here we can observe the cyclic pattern for mortality rates as well.

Despite its cyclic nature as observed in the time series, pertussis incidence, hospitalizations and mortality rates in the overall population increased dramatically, beginning in 2011 and peaking in 2014, well beyond prior peaks in the study period (Fig. 1).

Pertussis mortality rate in 2000 was unexpectedly high (0.016/100,000 population), corresponding to an outbreak in one state (Roraima, Northern Region) which recorded 18% (n = 5) of the total pertussis deaths (n = 27) in the country in this year. A total of 85 cases were confirmed in the State during the outbreak, and all of the deaths occurred in children younger than 6 months. Of note, DTP3 coverage in Roraima in 1999, the year prior to the outbreak was 73%, reflecting that the outbreak occurred in a low vaccine coverage setting.

Of interest, proportion of pertussis cases confirmed by laboratory over time increased progressively over time from 5% in 2002 to 30% in 2008, and then ranging from 23 to 35% each year in 2010–2016 (23% in 2009, 42% in 2010, 50% in 2011, 37% in 2012, 34% in 2013, 36% in 2014, 26% in 2015, and 22% in 2016).

### 3.2 Pertussis burden by age group

The vast majority of confirmed cases in the study period are observed in children under 5 years of age (78%), while 92.6% of all cases recognized in individuals <20 years of age. Notably, most of the disease burden occurred in children younger than 1 year of age, especially its more serious outcomes, with 62.5% percent of cases and 87.6% percent of hospitalizations. Deaths are concentrated in children younger than 6 months of age (94%) (Table 1).

Of interest, the number of pertussis hospitalizations reported to SIH in children younger than 2 months of age (7860) is higher than the number of all pertussis cases reported to SINAN in this same age group (7298). This was not expected, as SIH only reports hospitalizations occurring in the public health system (SUS) and therefore do not include hospitalizations in the private health care service sector. On the contrary, one would expect that hospitalized cases reported to SIH would capture only a fraction of the total cases hospitalized in the country. This is not the case with SINAN and SIM data as they are unrelated to the type of healthcare system providing care. This result may be related to the fact that hospitalization records are likely to be more complete as these information are used for reimbursement purposes.

**Fig. 2:**
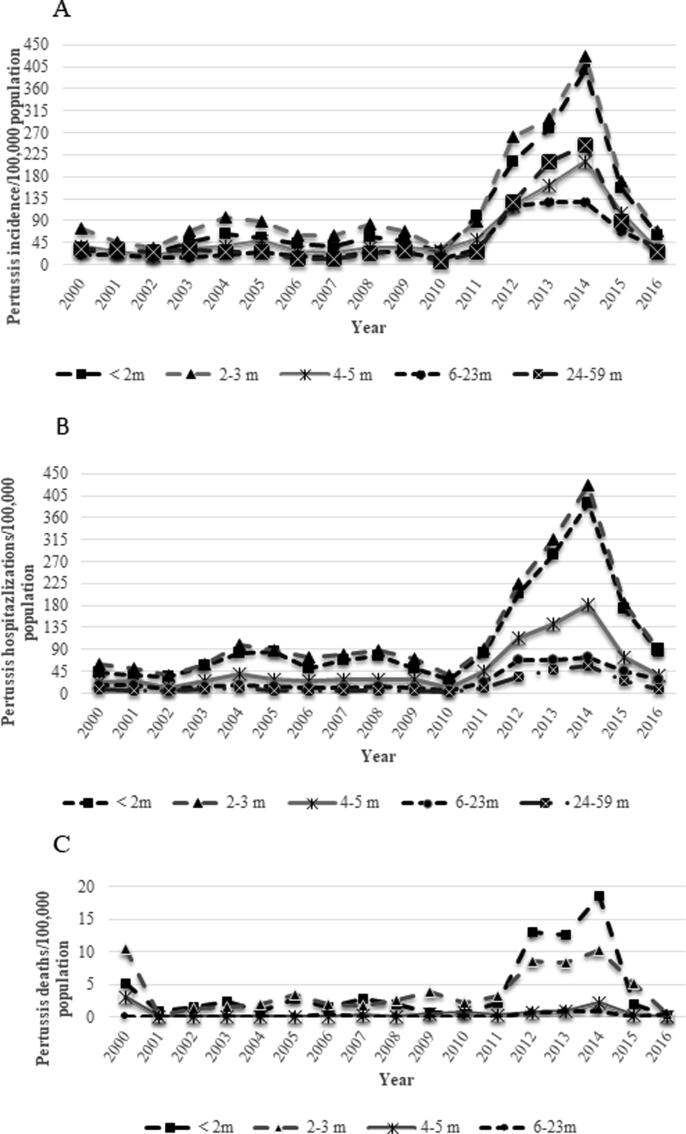
Pertussis rates/100,000 population by age sub-groups in children under 5 years in Brazil, 2000–2016. (A) Pertussis incidence rates by age sub-groups in children under 5 years; (B) Pertussis hospitalization rates by age sub-groups in children under 5 years; (C) Pertussis mortality rates by age sub-groups in children under 2 years. Brazil, 2000–2016.

When incidence rates by age groups are analyzed over time, it is possible to observe that the highest incidence is concentrated in children younger than 4 months, with much higher risk in children under 5 years of age when compared to older age groups (Fig. 2). In 2011 incidence rates begin to increase in all age groups.

Beginning in 2011, incidence rates double in all age groups. It is evident that pertussis risk is higher in children younger than 5 years of age, when compared to older age groups. Of interest, distribution of rates by age group remained similar as the disease incidence increased in the outbreak period, with higher rates in children younger than 4 months of age followed by those aged 4–5 months. Children younger than 4 months are those with incomplete immunization schedule, so this is expected. Rates in children aged 6–23 and 24–59 months did not increase significantly in the outbreak period (Fig. 2A).

Hospitalization rates are also higher in children under 6 months of age, particularly those younger than 4 months of age. For children aged 6–23 and 24–59 months of age, hospitalization rates did not increase significantly, as observed for incidence rates (Fig. 2B).

Over the years the highest mortality rate is observed in children younger than 4 months, especially those under 2 months of age (Fig. 2C). Mortality rates during the outbreak period are significantly higher in children under 2 months of age, followed by those aged 2–3 months of age, when compared to other age groups (Fig. 2C). This reinforces the fact that deaths are most likely to occur in children too young to be vaccinated, both prior and during the outbreak period.

### 3.3 Pertussis burden by geographical region and by outbreak period

When analyzing across geographical macro-regions in the country and comparing the pre-outbreak (2000–2010) to the outbreak (2011–2016) periods, significant increase in incidence rates was observed in all age groups in all regions except for the Northern Region, in which this increase is only significant in children <2 month of age. Pre-outbreak incidence rates were lower in the Northeastern region, in which Bahia state is located (Table 2).

**Table 2:** Pertussis incidence rates (per 100,000 population) in children under 5 years, by macro-region and age groups, during the pre-outbreak (2000–2010) and outbreak (2011–2016) periods. Brazil, 2000–2016.

**Region**	**Age groups**	**2000**–**2010**	**2011**–**2016**	**p-value**
		**Average incidence rates (95% confidence interval)**		
North	<2 m	27.9 (17.3–40.6)	88.8(48.9–131.6)	<0.05
	2–3 m	58.2 (43.8–73.3)	102.3 (56.6–149.1)	0.32
	4–5 m	34.9 (26.1–42.3)	64.1 (38.6–90.2)	0.07
	6–23 m	8.2 (5.6–10.6)	11.6 (6.6–15.8)	0.21
	24–59 m	3.9 (2.5–5.3)	3.5 (1.3–6.1)	0.76

Northeast	<2 m	26.9 (19.6–34.3)	170.9 (89.5–298.8)	<0.05
	2–3 m	35.2 (26.2–43.5)	176.4 (74.6–337.3)	<0.05
	4–5 m	16.5 (12.2–21.7)	89.5 (40.8–163.1)	<0.05
	6–23 m	2.4 (1.7–3.1)	11.3 (5.5–20.2)	<0.05
	24–59 m	0.7 (0.4–1.0)	4.0 (1.6–7.7)	<0.05

Southeast	<2 m	37.6 (28.1–47.6)	246.2 (109.9–361.2)	<0.05
	2–3 m	43.2 (31.8–55.6)	276.3 (144.5–409.7)	<0.05
	4–5 m	17.9 (13.8–22.3)	120.6 (65.4–173.9)	<0.05
	6–23 m	1.8 (1,3–2.6)	17.9(8.3–27.9)	<0.05
	24–59 m	0.4 (0.2–0.5)	6.9 (2.2–11.5)	<0.05

Midwest	<2 m	52.8 (40.2–66.5)	231.3 (85.3–373.0)	<0.05
	2–3 m	77.1(63.3–90.9)	217.1 (78.9–363.2)	0.10
	4–5 m	33.6 (25.5–42.2)	105.8(41.9–182.1)	0.08
	6–23 m	3.8 (2.6–5.1)	13.6 (5.3–23.2)	<0.05
	24–59 m	0.7 (0.4–1.1)	4.2 (0.8–8.4)	0.12

South	<2 m	63.9 (40.9–84.9)	293.4 (138.8–448.2)	<0.05
	2–3 m	94.54 (64.8–128.2)	367.1 (184.3–571.3)	<0.05
	4–5 m	46.73 (32.6–63.8)	180.9 (91.1–270.7)	<0.05
	6–23 m	5.2 (3.5–6.9)	29.9 (14.4–45.9)	<0.05
	24–59 m	1.1 (0.7–1.6)	8.8 (3.2–16.1)	<0.05

When looking at the state level, pertussis incidence rates in children <6 months of age increase dramatically in all states between 2012 and 2014. In some states the incidence levels reached higher peaks, and the year in which the incidence peaked varies from state to state (Table 6). São Paulo and Paraná states, neighboring states in the Southeast and south Region, are such states, reaching incidence rates higher than 500/100,000 children in 2013 and 2014 respectively.

A significant increase in hospitalization rates was also observed for all age sub-groups in children under 5 years in all macro-regions of the country during the outbreak period. Pre-outbreak hospitalization rates were also lower in the Northeastern region (Table 3).

**Table 3:** Pertussis hospitalization rates (per 100,000 population) in children under 5 years, by macro-region and age groups, during the pre-outbreak (2000–2010) and outbreak (2011–2016) periods. Brazil, 2000–2016.

**Region**	**Age groups**	**2000**–**2010**	**2011**–**2016**	**p-value**
		**Average hospitalization rates (95% confidence interval)**		
North	<2 m	29.7 (19.3–40.3)	95.2 (44.4–148.6)	<0.05
	2–3 m	41.4 (27.6–56.4)	126.1 (64.8–191.8)	<0.05
	4–5 m	21.5 (13.2–30.1)	81.6 (43.6–126.1)	<0.05
	6–23 m	3.0 (2.2–3.9)	9.8 (6.1–13.1)	<0.05
	24–59 m	0.6 (0.4-0.8)	1.5 (0.8–2.3)	0.03

Northeast	<2 m	40.3 (27.9–52.4)	194.3 (101.6–329.3)	<0.05
	2–3 m	41.7 (30.8–52.3)	184.2 (77.0–336.4)	<0.05
	4–5 m	17.2 (12.2–19.9)	80.6 (38.6–144.8)	<0.05
	6–23 m	1.9 (1.2–2.5)	7.7(4.8–11.4)	<0.05
	24–59 m	0.3 (0.2–0.3)	1.3 (0.7–2.0)	<0.05

Southeast	<2 m	52.9 (43.3–63.5)	235.9 (134.6–319.0)	<0.05
	2–3 m	59.6 (47.0–72.3)	225.6 (139.1–317.1)	<0.05
	4–5 m	18.2 (13.4–23.2)	91.4 (56.6–126.2)	<0.05
	6–23 m	1.3 (0.9–1.7)	9.8 (6.5–13.2)	<0.05
	24–59 m	0.1 (0.1–0.2)	1.2 (0.5–2.1)	<0.05

Midwest	<2 m	75.2 (55.8–97.5)	234.1 (112.4–361.2)	<0.05
	2–3 m	80.0 (65.9–95.0)	234.8 (105.7–397.1)	<0.05
	4–5 m	24.2 (17.2–30.5)	74.9 (32.3–128.1)	<0.05
	6–23 m	2.4 (1.8–3.1)	8.0 (3.7–12.1)	<0.05
	24–59 m	0.3 (0.1–0.4)	1.2 (0.6–1.9)	<0.05

South	<2 m	78.7 (63.4–95.3)	239.5 (111.6–364.2)	<0.05
	2–3 m	104.5 (84.9–122.5)	264.9 (151.7–402.5)	<0.05
	4–5 m	41.3 (31.3–50.4)	135.9 (66.6–205.4)	<0.05
	6–23 m	4.1 (3.3–4.9)	15.3 (7.6–23.7)	<0.05
	24–59 m	0.5 (0.3–0.7)	2.4 (0.7–4.0)	0.06

Significant increase in mortality rates was observed in children <2 months and 2–3 months of age in the South Region and in these plus 4–5 months in the Southeast Region (Table 4).

**Table 4:** Pertussis mortality rates (per 100,000 population) in children under 2 years, by macro-region and age groups, during the pre-outbreak (2000–2010) and outbreak (2011–2016) periods. Brazil, 2000–2016.

**Region**	**Age groups**	**2000**–**2010**	**2011**–**2016**	**p-value**
		**Average mortality rates (95% confidence interval)**		
North	<2 m	1.6 (0.5–3.1)	6.2 (0.8–11.5)	0.14
	2–3 m	4.2 (2.2–6.4)	6.8 (1.8–11.7)	0.27
	4–5 m	1.0 ((0.4–1.7)	0.8 (0.00–2.0)	0.70
	6–23 m	0.0 (0.0–0.1)	0.2 (0.0–0.3)	0.48

Northeast	<2 m	1.7 (0.9–2.5)	5.8 (2.0–10.7)	0.11
	2–3 m	1.3 (0.9–1.8)	4.1 (1.0–7.9)	0.15
	4–5 m	0.3 (0.0–0.6)	1.6 (0.2–3.2)	0.05
	6–23 m	0.0 (0.0–0.1)	0.1 (0.0–0.2)	0.26

Southeast	<2 m	1.3 (0.7–2.0)	9.2 (3.0–14.7)	<0.05
	2–3 m	1.2 (0.8–1.5)	7.1 (3.9–9.9)	<0.05
	4–5 m	0.1(0.0–0.3)	0.9 (0.4–1.5)	<0.05
	6–23 m	0.0 (0.0–0.0)	0.1 (0.0–0.2)	0.19

Midwest	<2 m	3.0 (1.5–4.8)	14.8 (2.8–31.2)	0.10
	2–3 m	1.3 (0.2–2.8)	6.5 (2.2–12.5)	0.12
	4–5 m	0.2 (0.0–0.7)	0.9 (0.0–3.0)	0.36
	6–23 m	0.0(0.0–0.0)	0.1 (0.0–0.2)	0.17

South	<2 m	1.8 (0.8–3.0)	8.11(2.6–14.9)	0.07
	2–3 m	1.8 (0.8–2.8)	5.38 (3.3–8.4)	<0.05
	4–5 m	0.0 (0.0–0.0)	0.31 (0.0–1.1)	0.36
	6–23 m	0.0 (0.0–0.5)	0.1 (0.0–0.2)	0.16

When looking at the increase in pertussis incidence rates in children <6 months of age during the peak of the outbreak period (2011–2014) and considering 2000 and 2010 as baseline pre-outbreak years, we can observe that the peak of the outbreak occurred in 2014 in most states. However, variations on the magnitude of the variation, the year of increase and peak and the relationship between incidence, hospitalization and mortality rates can be observed across different states. Reported incidence does not seem to be related with reported DTP3 coverage in children in the study period. Maternal Tdap vaccination was only introduced in Brazil in late 2014 so coverages are still low in 2015 and 2016 (Table 5).

**Table 5:** Vaccine coverage in children (DTP3) and pregnant women (Tdap), by states, and selected years during the pre-outbreak (2000, 2010) and outbreak (2011–2016) periods, Brazil.

State	**2000**	**2010**	**2011**	**2012**	**2013**	**2014**	**2015**		**2016**	
	DTP3 in children <1 year	DTP3 in children <1 year	DTP3 in children <1 year	DTP3 in children <1 year	DTP3 in children <1 year	DTP3 in children <1 year	DTP3 in children <1 year	Maternal Tdap	DTP3 in children <1 year	Maternal Tdap
**Midwest**										
Federal District	99.4	91.9	85.8	93	100.6	90.1	66.4	53.3	147.1	7.3
Goiás	96.9	102.3	102.1	83.7	100.1	87.7	94.9	21.1	94.5	10.3
Mato Grosso do Sul	98.8	100.7	98.7	95.4	111.4	122.9	117.9	43.7	105.2	50.6
Mato Grosso	101.1	97	94	90.6	94.6	92.1	101.7	43.8	104.8	40

**North**										
Acre	65	94.4	91.8	88.5	85.5	63.1	81.2	12.1	93.3	16.5
Amazonas	78.6	96	90.7	91.7	83	85.5	94.6	48.8	100.7	8.2
Amapá	85.7	89	85.1	83.7	87.8	71.4	84.6	49.2	106	43.2
Pará	84.7	102.8	96.6	92.6	91.2	80	73.1	7.2	78.8	3.9
Rondônia	101.7	103.8	101	102.9	99.4	101.7	104.5	65.8	111.1	58.3
Roraima	129	94.6	93	76.9	75.6	82.6	96.7	43.7	95.2	45.4
Tocantins	108.5	99.3	100.3	91.4	101.9	91.8	98.7	27.3	98.3	3.8

**Northeast**										
Alagoas	90.9	101.6	92.6	92.1	95.1	91.3	91.4	44.6	94.9	43
Bahia	97.4	99.2	100	97.2	96.2	94.5	93	43	81	34.1
Ceará	117.8	103.5	102.4	97.2	101.2	97.5	106.7	58.1	121.7	52.2
Maranhão	97.5	108	102.5	92.4	102.3	88.3	91.7	47.4	84.8	46.1
Paraiba	111.3	103.7	104.1	93.2	101.5	91.9	93.6	42.2	93.7	44.8
Pernambuco	94.5	106.3	108.6	100.2	97.2	97	103.6	63.7	105.9	49.9
Piauí	94.8	100	101.1	98.9	97.8	82.6	82	17.5	84	20.2
Rio Grande do Norte	88.3	97.7	98.8	97.7	90.5	87.3	90.6	43.5	85	41.2
Sergipe	100.5	102.8	103.8	100.1	101.2	93.3	93.9	44.9	91.1	17

**Southeast**										
Espirito Santo	100.1	100.4	102.8	99.2	95.7	93.4	99.6	56.6	99.3	38.4
Minas Gerais	115.7	100.4	101.2	94.7	103.3	89.4	100.7	42.1	100.9	23.1
Rio de Janeiro	91.2	92.8	95.8	88.8	91.1	90	99.3	54.6	104.6	47.1
São Paulo	100.2	95.4	96.8	91.9	97.7	94.1	98.4	61.1	93.5	16.6

**South**										
Paraná	103.9	98.3	101.1	97.1	101.2	95.4	101.3	30.8	97.9	2.1
Rio Grande do Sul	97.5	92.4	91.9	82.4	98.7	91.9	91.3	23.9	93.3	15.2
Santa Catarina	103.1	97.7	97.6	93.2	94.7	93.7	104.8	51.5	97.1	48.5

**Brazil**	99.7	98.7	98.5	93.1	97.4	91.9	96.3	48.4	96.8	30.3

DTP3-Coverage DTP third dose, Tdap-Coverage Tdap in pregnant.

Considering concentration of pertussis hospitalization and mortality is in children <6 months of age, we focus the analysis by state level in this age group (Table 6). Highlighted below the states considered in the state level dynamic transmission models reported by Luz et al [4].

In addition to different socio-economic conditions, theses 3 states present varying conditions of disease epidemiology, surveillance, and pertussis vaccine coverage in children. The two states with higher incidence rates during the outbreak period were also São Paulo and Paraná, despite higher vaccine coverages in children. On the other hand, Bahia state, located in the Northeast Region, had the lowest pre-outbreak disease burden (Table 6)), and lower vaccine coverages (Table 5). Nonetheless, disease burden peaked later during the 2011–2014 outbreak, and mortality rates did not increase as much.

**Table 6:** Pertussis incidence, hospitalization and mortality rates (/100.000 population) in children under 6 months, by states, and selected years during the pre-outbreak (2000, 2010) and outbreak (2011–2014) periods, Brazil.

	**2000**			**2010**			**2011**			**2012**			**2013**			**2014**		
State	Incid	Hosp	Mort	Incid	Hosp	Mort	Incid	Hosp	Mort	Incid	Hosp	Mort	Incid	Hosp	Mort	Incid	Hosp	Mort
**Midwest**																		
Federal District	54.1	142.7	0	76.9	110.6	0	112	272.1	0	247.6	402.1	22.6	489.7	456.7	17.2	931.3	637.2	23.6
Goiás	81.3	46.1	2.2	26.2	21.3	0	9.7	21.5	2.4	107.1	97.7	0	233.6	169.6	2.5	281.7	289.8	30.1
Mato Grosso do Sul	45.7	20.5	5.3	51.4	71.9	0	135.1	67.9	5.2	286.2	202.2	5.2	380.5	406.3	26.5	451.2	519.8	15.8
Mato Grosso	8.1	16.1	4.1	0	4.3	0	0	8.5	0	63.2	63.4	0	217.9	106.3	8.8	260.6	294.2	30.3

**North**																		
Acre	27.7	27.1	0	0	0	13.6	152.4	152.1	0	60.9	60.1	15.2	42.5	43.5	0	304.3	202	27.3
Amazonas	34.7	16.1	5.5	6.1	6.1	3	36.7	3.1	0	274.6	199.4	22	208.6	139.4	6.4	99.1	51.1	3.2
Amapá	240.6	64.2	0	16.3	16.3	0	0	111.8	0	65.3	271.2	0	238.4	622.7	16.4	384.1	1037.3	0
Pará	34.6	16.3	4.1	18.4	9.2	1.6	17.2	15.6	1.6	28.2	42.4	1.6	31.9	73.5	1.6	96.7	163.2	11.2
Rondônia	34	34.6	0	108.5	41.6	0	163.1	60.2	0	34.8	60.4	0	214.5	240.9	18.2	374.5	310.1	18.6
Roraima	293.8	22.7	121.1	23.3	0	0	94.2	118	0	141.5	164.9	23.5	352.8	682.8	23.8	93.7	165.4	0
Tocantins	0	38.4	0	0	8.3	0	8.4	8.4	0	44.4	71.5	0	106.9	249.9	0	459.9	495.6	9.3

**Northeast**																		
Alagoas	23.2	23.8	0	26.4	30.4	3.8	43.3	30.9	0	63.4	75.4	11.7	309.1	231.5	8.1	462.4	291.9	8.1
Bahia	20.3	29.4	0	14.7	18.2	0.9	78.2	91.1	0	71	114.1	1.1	118.4	141.2	3.3	302.4	376	7.6
Ceará	2.6	13.1	0	11.5	9.7	1.6	23.4	23.2	3.4	49.5	42.6	1.7	33.1	96	1.8	225.4	377.3	12.5
Maranhão	22.7	14.9	0	7.4	3.7	0	36.9	18.5	0	30	13.2	1.8	43.6	77.5	7.6	197.2	225.7	5.7
Paraiba	30.1	35.8	0	10.6	3.4	3.6	10.5	10.4	0	31.9	63.8	10.7	79.6	180.3	7.2	94.7	192.8	0
Pernambuco	108.2	178.5	3.7	32.7	37.8	1.6	88.7	60.6	6.5	322.7	118.6	8.2	173.2	243.6	6.8	953.5	761	22
Piauí	14.2	3.5	0	4.4	8.8	0	0	4.5	0	23.3	4.6	0	223.2	23.7	0	568.1	92.3	0
Rio Grande do Norte	14	21.4	3.4	39.5	47.1	0	181.5	175.9	8.6	332	573.2	4.4	300.2	389	4.5	262.1	309	9.2
Sergipe	24.6	4.9	0	0	12.1	0	25.3	37.1	0	12.6	87.7	0	58.4	151.3	0	72.2	390.8	7

**Southeast**																		
Espirito Santo	99.6	60	12.1	44.3	10.6	0	122.9	64.4	0	1078.8	477.6	18.3	990.7	283.8	0	397	179.3	0
Minas Gerais	6.4	68.8	0	13	26	0	44	51	1.6	162.3	172	2.6	215.5	245	6.2	180.9	226.9	6.4
Rio de Janeiro	7.4	34.4	1.7	18.9	16.9	1.1	136.1	88.5	6.5	171.2	209.5	13.4	96.2	124.8	3.4	88.5	116.7	1.2
São Paulo	15.3	46.3	0.3	46.7	35.8	3.1	241.6	149	5.5	263	224.8	8	376.4	281.1	11.3	520.7	409.2	14.7

**South**																		
Paraná	2.4	24.7	2.4	13.8	5.5	0	141.5	45.2	2.8	322.3	116.4	11.6	403.2	162.2	11.7	750.8	281.3	12
Rio Grande do Sul	4.8	84	0	116.6	63	0	153.8	149	3.2	659.1	584.3	8.3	414	532.1	3.4	239.1	219.7	0
Santa Catarina	15.3	19.7	2.2	36.6	53.7	0	96.2	123.3	4.9	381.5	393.9	7.5	297.4	322.6	0	249.7	372	2.5

Incid = incidence rate; Hosp = hospitalization; rate Mort = mortality rate.

## 4 Discussion

We describes the dynamics of pertussis over a long time series in Brazil, a middle income country with a strong national immunization program in which wP is routinely administered to infants. Brazil has robust national registries and health information systems which have been used to assess the impact of various immunization interventions [18], [19], [20]. We took the advantage of various information systems and analyzed state level data over time, considering reported cases, hospitalizations and mortality in the country as a whole and each of its 27 different states, including their features and patterns.

We present the epidemiologic data used to build the pertussis dynamic transmission model which is further described by Sun-Young[3] and Luz et al [4] in this issue, in a scenario of resurging pertussis that has become a problem in many LMICs.

Pertussis resurgence in Brazil had already been demonstrated previously in the national level [21] and in the state of São Paulo [22]. Most of the available studies on pertussis disease burden in MIC, including 2 studies conducted in Brazil, considered only a single data source [21], [22], [23], [24], [25]. A few studies reported in deaths and reported cases [26], [27]. Considering the limitations of secondary health information data sources, by using data from a variety of information systems, we were able to have a better understanding of pertussis burden in a LMIC, including different states with varying socio-economic and epidemiologic conditions.

Data of pertussis cases reported to the national surveillance system, pertussis patients hospitalized within the public healthcare system, and deaths due to pertussis during a period of 16 years (2000–2016) demonstrate that, in addition to the periodic increases of cases due to its cyclical nature of the disease, a resurgence of cases over and above the expected occurred in 2011 peaking in 2014. This was consistently observed for outpatient cases (8550 reported pertussis cases), hospitalized cases (4931 pertussis hospitalizations) and pertussis deaths 133 deaths due to pertussis), obtained from three different information systems. It was also observed in the country as a whole, although some states peaked earlier. Overall disease burden was most significant in children <1 year of age, with children younger than 6 months at higher risk for hospitalization and death. This is maintained throughout time, even during the most recent epidemic.

A systematic review on pertussis epidemiology in Latin America and the Caribbean Region Countries (LAC) showed that pertussis incidence estimates for the period 1980–2000 (17.8 cases/100 000 population (95% CI: 5.9–29.7)); was significantly higher than after 2000 (2.5 cases/100 000 population (95% CI: 1.8–3.2) [23]. This decline is likely attributable to the widespread introduction of pertussis vaccines during the 1980 s. Initial reports of disease resurgence in developed countries using acellular pertussis (aP) such as the United States peaking in 2000 [28], Canada with outbreaks in 2008 and 2012 [29], and in the Netherland in 2012 [30], [31] led to the hypothesis that these outbreaks were related to aP efficacy. Nonetheless, outbreaks in various developing countries using wP were also reported. Diminishing coverages have also been hypothesized to be associated to the resurgences. Nonetheless, DTP3 vaccine coverage estimates in LAC countries are reportedly increasing over the last 4 decades, with 72.4% coverage in 1980–1990 (95%CI: 64.6–80.2%); 79% in 1991–2000 (95% CI: 66.1–91.9%), and 90% in 2001–2015 (95% CI: 87.7–92.3%) [23]. Although Latin American countries reports high vaccine coverages, a dramatic increase in cases and deaths were reported by several countries in the Region including [32], [24], Argentina [26], and Colombia [23].

Another hypothesis has been supported by recent evidence from Peru [24], where authors have associated the increased disease burden with high prevalence two emerging genotypes of Bordetella pertussis.

Most studies report the most significant disease burden in children under 1 year of age, and particularly in younger than 6 months for more severe disease. When looking closer at disaggregated age groups, we observed the same pattern, corroborating the available evidence on the importance of this age group, which was the main target population for the strategies considered in our models. However, during the outbreak, a significant increase in risk of disease in 6–23 months and 24–59 months of age was also observed. One may hypothesize that this can be related to lower vaccine coverages or even more likely, delayed vaccination of the 3rd dose of the wP primary schedule recommended at 2, 4 and 6 months of age, both of which have been reported by studies evaluation vaccine coverage in Brazil [21], [22].

Brazil is large country with important socio-economic inequalities across its 27 States, with lower average per capita GDP concentrated in its Northern and Northeastern regions. The country’s per capita income is GDP R$19,7 mi, ranging from R$ 6.9 to R$ 58.5mi 6.8 and among the various. The same is observed for HDI across the States, varying from 0.631 to HDI: 0.824 across States.

The two states with higher incidence rates during the outbreak period (São Paulo and Paraná) and one state in the Northeast Region (Bahia) with the lowest pre-outbreak disease burden, were selected for the 3-states dynamic model reported by Luz et al [4]. Differently than other Regions, in the Northeast Region (were Bahia state in located) mortality rates increased also in children aged 4–5 months. In the Southeast Region (were Parana and São Paulo state are located), significant increase in rates can be observed in both 4–5 and 6–23 months of age children. Paraná and São Paulo are also among the states with better surveillance [33]. These states were among the first ones to implement qPCR-based laboratory confirmation of pertussis cases in 2009 in São Paulo and 2014 for the state of Paraná. Furthermore, special hospital based surveillance sites were implemented in these states between 2000 and 2015, sentinel sites function to improve both the quality of the epidemiology variables and the quality of the collection and transport of specimens to the reference laboratory of the respective state, Paraná has five sentinel centers and São Paulo with 33 [33]. Variability in other aspects such social, demographic, climatic can also be observed by state and Region. In the south of the country where is the Paraná State, the weather is colder (subtropical climate) compared to other regions, but the great majority of the regions are warm, having some places with semi-arid climate in the Bahia State and others. Regarding the distribution of the population, the State of São Paulo has the biggest demographic density of the country (166.23 inhabitants / km), concentrating around 50% of the population under 5 years of the Southeast region.

As such, by using Brazilian robust data from a variety of independent sources, and stratifying further by geographical location and age groups, we could adequately model disease burden and transmission in a variety of settings representing other LMIC countries

Nonetheless, the use of these data incurred several in limitations worthwhile noting. Reported cases are likely to be under reported as many cases are sub-clinical and do not reach healthcare services. Changes in laboratory confirmation method used, improvements in the surveillance system, and changes to the SINAN database platform may also have impacted the estimates of confirmed pertussis cases reported to SINAN over time. Hospitalization records are likely to be more complete, although part of the population using private healthcare system are not reported to SIH, which required us to make assumptions, for modelling purposes, that similar rates of hospitalization would occur in both populations. Finally, despite the fact that mortality information systems has evolved in the past decades, being likely that coverage and quality of the data has improved over years, under-registration of deaths persists in some Brazilian states, mainly in the North and Northeast, where death declarations may have been underestimated.

In conclusion, as pertussis is major cause of global morbidity and mortality in young children, especially in LMICs, alternative strategies for the mitigation of its burden are being evaluated, and are mostly needed in countries with the highest burden. Such countries are less likely to have reliable data on disease burden and epidemiology, and as so, Brazil, being a large middle income country with heterogeneous conditions, with robust long-term data on pertussis morbidity and morbidity, has provided reliable data required to build and calibrate dynamic transmission models which were used to evaluate the cost-effectiveness of maternal immunization, considering low- and middle income country scenarios.

## Funding

This work was supported by the Bill & Melinda Gates Foundation, Seattle, WA [grant number OPP1124529]. CMT (#308010/2018–3) receive scientific productivity scholarships from the Brazilian National Council for Scientific and Technological Development (CNPq).

AB is a scholar with grant from the Coordenação de Aperfeiçoamento de Pessoal de Nível Superior (CAPES) (Finance Code 001).

## CRediT authorship contribution statement

**Angela M. Bagattini**: Formal data analysis, Validation, Writing - original draft, Writing - review & editing. **Gabriela Policena**: Methodology, Data curation. **Ruth Minamisava**: Methodology, Data curation. **Ana Lucia S. Andrade**: Methodology, Data curation. **Sérgio de A. Nishioka**: Methodology, Data curation. **Anushua Sinha**: Conceptualization, Funding acquisition, Project administration. **Louise B. Russell**: Conceptualization, Funding acquisition, Project administration, Formal data analysis, Validation. **Cristiana M. Toscano**: Conceptualization, Funding acquisition, Project administration, Supervision, Formal data analysis, Validation, Writing - original draft, Writing - review & editing.

## Declaration of Competing Interest

The authors declare the following financial interests/personal relationships which may be considered as potential competing interests: ALA has received lecture fees and travel grants from GlaxoSmithKline and Pfizer; RM has received travel grants from GlaxoSmithKline. The other authors have no conflicts of interest.
