# Supplementary material for: The data used to build the models: Pertussis morbidity and mortality burden considering various Brazilian data sources
Source: Vaccine. 2021 Jan 3;39(1):137–46. doi: 10.1016/j.vaccine.2020.09.007 (PMC7738753; doi:10.1016/j.vaccine.2020.09.007)
Supplement: Supplementary data 1 [file mmc1.docx]

**Supplemental File 1: Maps and table of characteristics of the Brazilian states**

**Figure 1.** Map geopolitical division of the Brazilian territory by region. Brazil


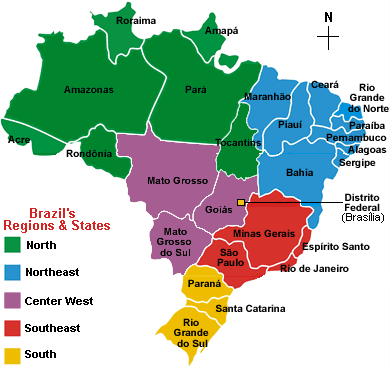


Source: Brazilian states Abbreviations & Information: <http://www.brazil-help.com/delta/brazilian_states.htm>

* Brazil is composed of the union of the 26 states and the Federal District.

**Table 1**. Characterization epidemiological and socio-demographic characteristics by state. Brazil, 2010.

| Macro-region/State | Total Population (inhabitants)* | Population density (inhab./km2)* | Coverage  .DTP 3 dose (%)* | Gini rate* | Infant Mortality Rate (under 1 year deaths per 1000 live births)** | Under-5 mortality rate (per 1,000 live births)** | HDI* | Per capita GDP (R$)* |
| --- | --- | --- | --- | --- | --- | --- | --- | --- |
| **Midwest** |  |  |  |  |  |  |  |  |
| Distrito Federal (DF) | 2,570,160 | 444.1 | 91.9 | 0.6 | 14.0 | 15.9 | 0.8 | 58,489.5 |
| Goiás (GO) | 6,003,788 | 17.7 | 102.3 | 0.6 | 14.0 | 16.3 | 0.7 | 16,251.7 |
| Mato Grosso (MT) | 3,035,122 | 3.4 | 97.0 | 0.6 | 16.8 | 20.3 | 0.7 | 19,644.1 |
| Mato Grosso do Sul (MS) | 2,449,024 | 6.9 | 100.7 | 0.6 | 18.1 | 21.5 | 0.7 | 17,765.7 |
| **North** |  |  |  |  |  |  |  |  |
| Acre (AC) | 733,559 | 4.5 | 94.4 | 0.6 | 23.0 | 24.9 | 0.7 | 11,567.4 |
| Amapá (AP) | 669,526 | 4.7 | 89.0 | 0.6 | 15.1 | 16.2 | 0.7 | 12,361.5 |
| Amazonas (AM) | 3,483,985 | 2.2 | 96.0 | 0.7 | 17.0 | 18.2 | 0.7 | 17,173.3 |
| Pará (PA) | 7,581,051 | 6.1 | 102.8 | 0.6 | 20.3 | 21.9 | 0.6 | 10,259.2 |
| Rondônia (RO) | 1,562,409 | 6.6 | 103.8 | 0.6 | 18.0 | 19.3 | 0.7 | 15,098.1 |
| Roraima (RR) | 450,479 | 2.0 | 94.6 | 0.6 | 16.1 | 17.2 | 0.7 | 14,051.9 |
| Tocantins (TO) | 1,383,445 | 5.0 | 99.3 | 0.6 | 19.6 | 21.1 | 0.7 | 12,461.7 |
| **Northeast** |  |  |  |  |  |  |  |  |
| Alagoas (AL) | 3,120,494 | 112.3 | 101.6 | 0.6 | 28.4 | 31.0 | 0.6 | 7,874.2 |
| Bahia (BA) | 14,016,906 | 24.8 | 99.2 | 0.6 | 21.7 | 23.5 | 0.7 | 11,007.5 |
| Ceará (CE) | 8,452,381 | 56.8 | 103.5 | 0.6 | 19.3 | 20.8 | 0.7 | 9,216.1 |
| Maranhão (MA) | 6,574,789 | 19.8 | 108.0 | 0.6 | 28.0 | 30.6 | 0.6 | 6,888.6 |
| Paraíba (PB) | 3,766,528 | 66.7 | 103.7 | 0.6 | 21.7 | 23.4 | 0.7 | 8,482.0 |
| Pernambuco (PE) | 8,796,448 | 89.6 | 106.3 | 0.6 | 20.4 | 22.0 | 0.7 | 10,821.6 |
| Piauí (PI) | 3,118,360 | 12.4 | 100.0 | 0.6 | 23.1 | 25.0 | 0.6 | 7,072.8 |
| Rio Grande do Norte (RN) | 3,168,027 | 60.0 | 97.7 | 0.6 | 19.7 | 21.2 | 0.7 | 10,207.6 |
| Sergipe (SE) | 2,068,017 | 94.4 | 102.8 | 0.6 | 22.2 | 24.0 | 0.7 | 11,572.4 |
| **Southeast** |  |  |  |  |  |  |  |  |
| Espírito Santo (ES) | 3,514,952 | 76.3 | 100.4 | 0.6 | 14.2 | 16.3 | 0.7 | 23,378.7 |
| Minas Gerais (MG) | 19,597,330 | 33.4 | 100.4 | 0.6 | 15.1 | 17.3 | 0.7 | 17,931.9 |
| Rio de Janeiro (RJ) | 15,989,929 | 365.2 | 92.8 | 0.6 | 14.2 | 16.3 | 0.8 | 25,455.4 |
| São Paulo (SP) | 41,262,199 | 166.3 | 95.4 | 0.6 | 13.9 | 15.9 | 0.8 | 30,243.2 |
| **South** |  |  |  |  |  |  |  |  |
| Paraná (PR) | 10,444,526 | 52.4 | 98.3 | 0.5 | 13.1 | 15.1 | 0.7 | 20,814.0 |
| Rio Grande do Sul (RS) | 10,693,929 | 39.8 | 92.4 | 0.5 | 12.4 | 14.3 | 0.7 | 23,606.4 |
| Santa Catarina (SC) | 6,248,436 | 65.3 | 97.7 | 0.5 | 11.5 | 13.4 | 0.8 | 24,398.4 |
| Brasil | 190,755,799 | 22.43 | 98.7 | 0.6 | 16.7 | 18.8 | 0.7 | 19,766.3 |

GDP: Gross Domestic Product; source of variables. HDI: Human Development Index

Data from the year 2010. the year preceding the outbreak.

*Source of variables: Brazilian Institute of Geography and Statistics (*Instituto Brasileiro de Geografia e Estatística – IBGE*); <http://www.brazil-help.com/delta/brazilian_states.htm>

** Source of variables: PNUD –United Nations Development Programme. IPEA- Institute for Applied Economic Research; João Pinheiro Foundation;. **Human Development Atlas in Brazil. 2013**; Brazilian Institute of Geography and Statistics (*Instituto Brasileiro de Geografia e Estatística – IBGE*). <http://www.atlasbrasil.org.br/2013/pt/consulta/> <http://www.atlasbrasil.org.br/2013/pt/consulta/>
